# The hormone-bound vitamin D receptor enhances the FBW7-dependent turnover of NF-κB subunits

**DOI:** 10.1038/srep13002

**Published:** 2015-08-13

**Authors:** Fatemeh Fekrmandi, Tian-Tian Wang, John H. White

**Affiliations:** 1 Departments of Medicine, McGill University, Montreal, Quebec, Canada; 2 Physiology, McGill University, Montreal, Quebec, Canada

## Abstract

Signaling by hormonal vitamin D, 1,25-dihydroxyvitamin D (1,25D) has attracted increasing interest because of its non-classical actions, particularly its putative anticancer properties and its role in controlling immune system function. Notably, the hormone-bound vitamin D receptor (VDR) suppresses signaling by pro-inflammatory NF-κB transcription factors, although the underlying mechanisms have remained elusive. Recently, the VDR was shown to enhance the turnover of the oncogenic transcription factor cMYC mediated by the E3 ligase and tumor suppressor FBW7. As FBW7 also controls the turnover of the p100 (NF-κB2) subunit of the family, we determined whether the 1,25D enhanced FBW7-dependent turnover of NF-κB subunits p100, p105 (NF-κB1) and p65 (RELA). Protein levels of all three subunits declined markedly in the presence of 1,25D in multiple cell lines in the absence of substantial changes in mRNA expression. The VDR coimmunoprecipitated with all three subunits, and 1,25D treatment accelerated subunit turnover in cycloheximide-treated cells. Importantly, we observed an association of FBW7 with p105 and p65, as well as p100, and knockdown of FBW7 eliminated 1,25D-dependent subunit turnover. Moreover, expression of NF-κB target genes was elevated in FBW7-depleted cells. These results reveal that 1,25D signaling suppresses NF-κB function by enhancing FBW7-dependent subunit turnover.

Vitamin D is obtained from limited dietary sources or photochemical and thermal conversion of 7-dehydrocholesterol in skin in the presence of adequate solar ultraviolet B radiation. It is 25-hydroxylated in the liver to produce the major circulating form and converted into hormonal 1,25-dihydroxyvitamin D3 (1,25D) in peripheral tissues^1^,^2^. 1,25D has many physiological actions in addition to its classic endocrine control of calcium and phosphate homeostasis^1^,^2^,^3^. The vitamin D receptor (VDR) is expressed very widely^3^, including in many cell types of the immune system, and 1,25D has anti-proliferative, pro-differentiating, anti-inflammatory, and immunomodulatory activities^1^,^4^,^5^,^6^,^7^. There is growing clinical evidence to support the non-classical actions of 1,25D^8^,^9^,^10^,^11^; epidemiological data have provided a correlation between the prevalence of certain cancers, as well as autoimmune conditions, and reduced exposure to sunlight^12^. 1,25D regulates gene transcription by binding to the VDR, which is a hormone-regulated transcription factor^1^,^3^,^13^,^14^. The ligand-bound VDR regulates gene transcription through direct DNA binding or interactions with other classes of transcription factors^15^,^16^,^17^, such as components of the nuclear factor kappa B (NF-κB) family^18^,^19^,^20^.

The NF-κB family consists of five members: NF-κB1 (p50 and precursor p105), NF-κB2 (p52 and precursor p100), RELA (p65), RELB and REL (c-Rel), which form homo- or heterodimers to promote inflammatory responses through two pathways^21^,^22^,^23^. NF-κB subunits are activated by inflammatory cytokines or innate immune signaling in immune cells and are critical for induction of inflammatory responses^24^. NF-κB signaling pathways are also often altered in human cancer, and pro-tumorigenic functions of the NF-κB canonical signaling pathway, through formation of p50:p65 heterodimers, are now confirmed^25^. However, the non-canonical, p52/p100 (NF-κB2)-dependent pathway may also contribute to tumorigenesis^26^,^27^. Many malignant tumors such as those of the prostate exhibit increased levels and activity of NF-κB^28^. Pro-tumorigenic effects of NF-κB also occur in colorectal cancer, head and neck squamous cell carcinoma (HNSCC), glioblastoma, Hodgkin’s disease, hepatitis-associated hepatocellular carcinoma and multiple myeloma^29^,^30^. A tumor initiating microenvironment can arise in the context of chronic inflammation, for example in hepatitis or in colitis-associated cancer^31^,^32^,^33^. In this regard, NF-κB is considered to be a matchmaker between inflammation and cancer^29^, but further investigation is needed to understand the signals regulating NF-κB function under normal and pathophysiological conditions.

NF-κB inhibitors have become a focus of cancer research as blocking NF-κB activity prevents its tumor-promoting and pro-inflammatory functions^21^,^23^. The VDR can repress activation of NF-κB in various cell types via mechanisms that are poorly defined^34^,^35^. Intriguingly, recent studies showed that the non-canonical, p52/p100 (NF-κB2) pathway is inhibited via proteasomal turnover mediated by F-box protein FBW7, an E3-ubiquitin ligase and tumor suppressor^27^,^36^,^37^. Other work revealed that the VDR can regulate the FBW7-dependent proteasomal turnover of the oncogenic transcription factor cMYC and its antagonist MAD1/MXD1^38^. The SCF ubiquitin ligase complex that contains FBW7 (also known as FBXW7, CDC4, AGO, and SEL10) binds to and induces proteasomal degradation of several oncogenic proteins^39^,^40^,^41^. FBW7 controls the turnover of several proteins implicated in cell cycle progression and oncogenesis and its inactivation is associated with tumorigenesis^42^,^43^. FBW7 recognizes target proteins through consensus or near-consensus motifs called phosphodegrons^44^, with multiple motifs enhancing the affinity of FBW7-substrate interactions^45^,^46^.

We hypothesized that vitamin D signaling suppresses NF-κB function by stimulating FBW7-mediated turnover of its subunits and studied the role of the VDR and FBW7 in controlling target protein turnover of NF-κB subunits of both the canonical and non-canonical pathways. In multiple cancer cell lines, 1,25D treatment led to loss of protein expression of p100, p105 and p65 in the absence of significant regulation of their mRNAs. These effects were blocked in FBW7-depleted cells, revealing that 1,25D can suppress NF-κB function by enhancing the FBW7-dependent turnover of members of the family controlling both the canonical and non-canonical pathways.

## Results and Discussion

### Identification of 1,25D as a suppressor of NF-κB signaling pathways

**Figure 1:**
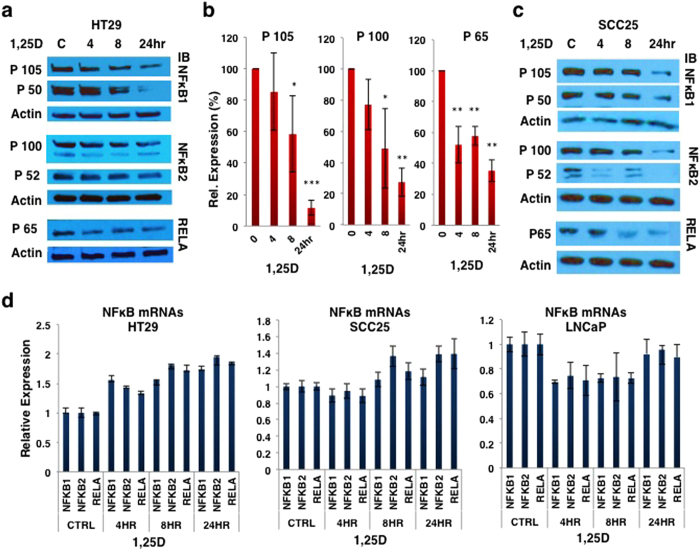
1,25D suppresses expression of different subunits of NF-κB protein. (**a**) Suppression of NF-κB protein expression in HT29 cells by 1,25D (100 nM) treatment. (**b**) Quantification of western blots of NF-κB expression in 1,25D treated HT29 cells from three different experiments; NF-κB1, NF-κB2, and RELA respectively. *P < 0.05, **P < 0.01, and ***P < 0.001 compared with paired control samples. (**c**) 1,25D suppresses expression of different subunits of NF-κB protein in SCC25 HNSCC cells. Note that the P100/P52 blot was stripped and reprobed for P65. (**d**) NF-κB mRNA expression after 1,25D treatment in HT29, SCC25 and LNCaP cells, as indicated.

To investigate the effect of 1,25D on NF-κB signaling pathways we used a series of human model cancer cell lines; the colon carcinoma line HT29, LNCaP prostate cancer cells, and SCC25 head and neck squamous carcinoma cells. Cells were treated with 1,25D (100 nM) for 24 h and protein expression of NF-κB1 (p105/p50) and RELA (p65), as key components of canonical pathway, and NF-κB2 (p100/p52), as the main component of non-canonical pathway^23^, was assessed by western blotting. Protein levels of all NF-κB subunits analyzed decreased in 1,25D-treated HT-29 (Fig. 1a,b), and SCC25 cells (Fig. 1c). A similar decline in expression of p105 and p65 was seen in 1,25D-treated LNCaP cells (Supplementary Fig. 1a). Loss of protein expression was not accompanied by substantial changes in mRNA levels (Fig. 1d), indicating that 1,25D was not suppressing transcription of the corresponding genes. Subsequent experiments were carried out in HT29 and LNCaP cell lines, as models for colorectal and prostate cancer, and in which the NF-κB pathway activation is known to promote tumorigenesis^29^.

### FBW7 modulates the NF-κB canonical pathway in a 1,25D dependent manner

**Figure 2:**
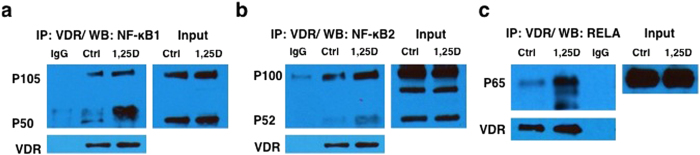
The VDR coimmunoprecipitates with NF-κB subunits. P105/P50 (**a**), P100/P52 (**b**), and RELA (**c**) from extracts of empty vehicle (Ctrl) or 1,25D-treated HT29 cells for 24 h.

To probe the mechanisms of 1,25D action, we examined the binding of NF-κB subunits to the VDR in coimmunoprecipitation (coIP) experiments followed by western blot analysis. We observed binding of all three NF-κB subunits with the VDR in extracts of HT29 cells (Fig. 2a–c). The association of the VDR with RELA was consistent with previous studies^47^. Note that, although we observed loss of NF-κB subunit expression in the presence of 1,25D (Fig. 1), the absence of 1, 25D-dependence of the protein-protein interactions observed was not unexpected, as previous work has shown that the VDR interacts constitutively with FoxO transcription factors and cofactors SIRT1 and protein phosphatase 1, but controls FoxO post-translational modification (deacetylation and dephosphorylation) and DNA binding in a 1, 25D-dependent manner^48^.

**Figure 3:**
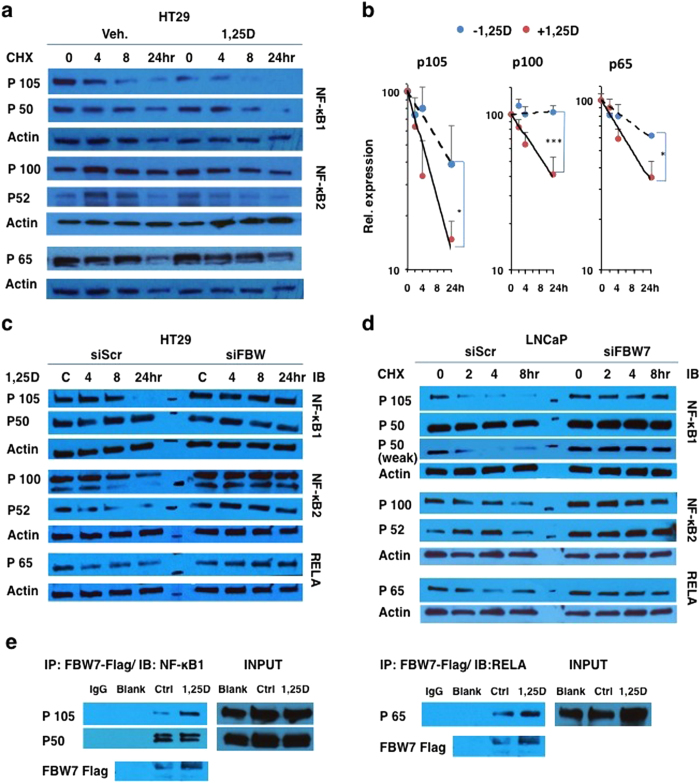
Ablation of FBW7 inhibits 1,25D-mediated turnover of NF-kB subunits. (**a**,**b**) 1,25D increases turnover of NF-κB subunits in cycloheximide-treated HT29 cells. Note that the P105/P50 blot was stripped and reprobed for P65. Quantification of data from 4 biological replicates is provided in (**b**). *P < 0.05, and ***P < 0.001 (**c**,**d**) Ablation of FBW7 eliminates NF-κB subunit turnover in 1,25D-treated HT29 cells (**c**) or in CHX-treated LNCaP cells (**d**). (**e**) Coimmunoprecipitation of NF-κB1 (left) and RELA (right) with flag-tagged FBW7 in HT29 cells.

To determine whether 1,25D treatment was altering protein turnover, cellular protein synthesis was blocked with cycloheximide (CHX) in HT29 cells after pre-treatment (4 h) with either vehicle or 1,25D (Fig. 3a,b). This revealed an accelerated loss of p105, p100 and p65 NF-κB subunits in 1,25D-treated cells relative to controls, results highly reminiscent of the effects of 1,25D on turnover of cMYC^38^. Similar results were obtained in LNCaP cells (Supplementary Fig. 1b). Recent work^36^,^37^ has shown that NF-κB2/P100 interacts with the E3 ubiquitin ligase FBW7 via consensus phosphodegron motifs, which promotes p100 degradation. As 1,25D treatment controls the FBW7-dependent turnover of cMYC and its antagonist MXD1^38^, we investigated the potential effects of FBW7 ablation on 1,25D-dependent turnover of RELA and NF-κB1 as well as NF-κB2 in HT29 cells. FBW7 knockdown completely abolished 1,25D-regulated p105, p100 and p65 turnover in these cells (Fig. 3c). In related experiments, FBW7 ablation abrogated loss of p105, p100 and p65 expression in cycloheximide-treated LNCaP cells (Fig. 3d), and blocked the turnover of p65 after 24 h of 1,25D treatment in LNCaP cells (Supplementary Fig. 2). The increased NF-κB2/p100 stability in FBW7-depleted cells was consistent with previous studies^27^,^36^,^37^, although NF-κB2 appeared to be the most stable of the subunits tested (Fig. 3a–d). The prolonged half-lives of p105, p65 and p100 observed in cycloheximide-treated cells after knockdown suggest that FBW7 is a physiological regulator of both the canonical pathway and non-canonical NF-κB pathways. To substantiate this hypothesis, coIP experiments were performed in cells transfected with FLAG-tagged FBW7 (note that available antibodies do not reliably recognized endogenous FBW7). This revealed the association of FBW7 with NF-κB subunits (Fig. 3e). Both p50 and p105 coimmunoprecipitated with tagged FBW7, the latter in a hormone-dependent manner. Given that the putative phospho-degron motifs of NF-κB1 are in the p105 portion of the protein (see below), it is not clear at this point the mechanism by which p50 associates with FBW7; this could occur through associations of p50 with a domain of FBW7 other than the substrate recognition domain, or through binding of FBW7 to a protein intermediate such as p50 heterodimeric partner p65. Note that, in control experiments, no signal was seen with immunoprecipitations in transfected cell extracts with control IgG or with anti-Flag antibody when extracts of untransfected cells were used (Blank; Fig. 3e).

**Figure 4:**
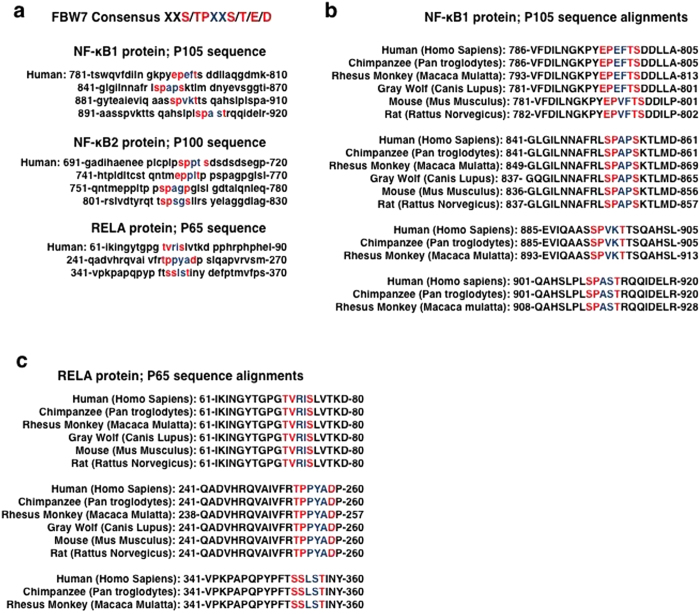
(**a**) Potential binding sites of FBW7 on NF-κB subunits in human. (**b**,**c**) Sequence alignment of NF-κB1 (**b**) or p65 (**c**) with the consensus FBW7 phosphodegrons in different species.

Taken together the results presented above indicate that the 1,25D-bound VDR promotes FBW7-dependent p105 and p65 degradation, and consequently decreases NF-κB canonical pathway activity. A similar effect of 1,25D was observed on the p100/p52 non-canonical pathway. FBW7 recognizes target proteins through a phosphodegron (core: S/TPxxS/T/E/D), although a number of substrates have been identified with near-consensus motifs^49^,^50^,^51^. Previous work showed that NF-κB2 contains a consensus phosphodegron in the p100 portion of the molecule, along with near consensus motifs^36^,^37^ (Fig. 4a). Examination of NF-κB1 and RELA protein sequences also revealed multiple consensus or near-consensus phosphodegron motifs (Fig. 4a). Notably, NF-κB1 contains near-consensus and consensus motifs within the C-terminal region of the protein unique to p105, including EPefTS, SPapS, SPvkT, and SPasT motifs. Many of these sites are evolutionarily conserved (Fig. 4b), suggesting that FBW7 recognizes both proteins as substrates. While the consensus sequence is recognized most strongly by FBW7, several targets have been identified that lack one or more elements of this motif. For example, the phosphodegron in MCL1 contains a hydrophobic residue instead of a proline^52^ similar to the TVriS sequence in p65 (Fig. 4a) , which is also widely conserved (Fig. 4c). In addition, motifs in mTOR, PGC1α and TP63 lack the downstream phosphorylation site^36^,^51^,^53^,^54^. Based on studies with other FBW7 target proteins^45^,^46^,^55^, it is likely that multiple motifs contribute to the association of FBW7 with NF-κB1 and p65.

### NF-κB subunit and target gene expression is enhanced in FBW7-depleted cells

**Figure 5:**
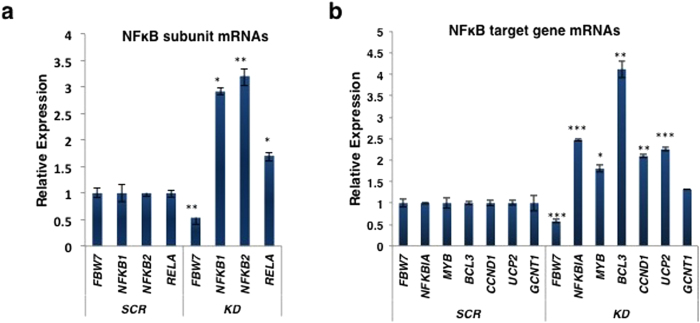
Increase in the mRNA expression of transcripts encoding NF-κB subunits (**a**) and NF-kB target genes (**b**) after FBW7 knockdown in LNCaP cells. *P < 0.05, **P < 0.01, and ***P < 0.001 compared with paired control samples.

If FBW7 expression was important for regulation of NF-κB function, one would predict that NF-κB transcriptional activity should be increased in FBW7-deficient cells. Gene expression analyses in LNCaP cells showed that after FBW7 knockdown there was an increase in the expression of transcripts encoding NF-κB subunits (Fig. 5a), consistent with autoregulation of NF-κB subunit gene transcription^56^,^57^. We also observed elevated mRNA expression of a number of NF-κB1/RELA transcriptional target genes after FBW7 knockdown (Fig. 5b), although that of *GCNT1* did not reach statistical significance.

These studies provide evidence that FBW7 controls the stability of components of both the canonical and non-canonical NF-κB pathways and provide a novel mechanism for 1,25D-mediated suppression of NF-κB function. We speculate that the VDR stabilizes the association of FBW7 with p105 and p65 enhancing their turnover. We predict that the domain of interaction of the VDR will not overlap critical phospho-degrons so as to preclude the formation of a VDR-FBW7-p105 or -p65 complex (diagrammed in supplementary Fig. 3). This mechanism is highly physiologically significant, as the collective results of several studies have shown that FBW7 functions as a tumor suppressor. Ablation of FBW7 in mice leads to tumorigenesis in a number of models^43^,^58^. More importantly, the inactivation of FBW7 has been observed in several human malignancies^42^,^59^, including HNSCC^60^,^61^, and 9% of colon cancers^62^, and many FBW7 mutations occur at hotspots encoding R465 and R479, which are critical for substrate recognition. In addition to NF-κB2, our findings add NF-κB1 and p65 to the list of known FBW7 target proteins. Other targets include cell cycle regulators such as cyclin E, cJUN, Notch^39^,^58^, and the p160 coactivator AIB1/ACTR/SRC3^63^, which is frequently overexpressed in a range of cancers and acts to drive cells into S phase^64^. Loss of FBW7 function may thus contribute to elevated NF-kB signaling in some malignancies.

## Methods

### Cell Culture

HT29 cells from the American Type Culture Collection (ATCC) were cultured in DMEM (319-005-CL; Wisent) supplemented with 10% FBS. LNCaP cells (ATCC) were cultured in RPMI-1640 (350-000-CL, Wisent) with 10% FBS. SCC25 (ATCC) were cultured in DMEM/F12 (319-085-CL, Wisent) with 10% FBS. Cells were treated with 100 nM 1,25D (679101, Calbiochem) or vehicle DMSO, as indicated.

### siRNA knockdowns

Cells were transfected with siRNAs (Qiagen; Supplementary Table 1) for 24 hours using Lipofectamine^TM^ 2000 (Invitrogen). Opti-mem I (1X) reduced serum medium (31985-062, Gibco^R^) was used for transfection.

### Plasmid Transfections

Cells were transfected for 24 hours using a Lipofectamine^TM^ 3000 transfection kit (Invitrogen). Plasmids containing Flag-FBW7 were prepared in-house. Opti-mem I (1X) reduced serum medium (31985-062, Gibco^R^) was used.

### RT-qPCR

Quantitative RT-PCR was performed with SsoFast-EvaGreen real-time PCR kit (Bio-Rad). Expression was normalized to *GAPDH*. Primer pairs used for RT-PCR were purchased from Invitrogen and are listed in Supplementary Table 2.

### Western blot and coimmunoprecipitation

Cells were lysed with lysis buffer 1 (20 mM Tris, pH 7.5, 100 mM NaCl, 0.5% Nonidet P-40, 0.5 mM EDTA, 0.5 mM EGTA). 4 μg anti-VDR (D-6; Santa Cruz) or Flag DYDDDK Tag Rabbit (2368S, Cell Signaling) antibodies were pre-bound for 2 hours to protein A agarose beads (SC-2001; Santa Cruz), washed with PBS plus 5% BSA and added to the lysate, followed by overnight immunoprecipitation. Protein A agarose beads were then washed 5x with washing buffer 2 (20 mM Tris, pH 7.5, 200 mM NaCl, 1% Nonidet P-40, 0.5 mM EDTA, 0.5 mM EGTA) and processed for Western blotting.

### Protein turnover studies

Cells were transfected with control or *FBW7* siRNAs using Lipofectamine^TM^ 2000 (Invitrogen), or treated with 1,25D, followed by incubation with cycloheximide (C7698-Sigma) at final concentration of 20 μg/mL. Cells were lysed with lysis buffer 1, followed by standard Western blotting.

### Antibodies

NF-κB1 (E-10), NF-κB2 (C-5), Actin (I-19), VDR (H-81) and VDR (D-6) were from Santa Cruz; NF-κB1 (3035S), DYDDDK Tag Rabbit (2368S) were from Cell Signaling; anti-NF-κB2 (2446848), and anti-NF-κB p65 (RelA; 62246) were from Millipore; and monoclonal anti-Flag M2 (F3165 & F804) from Sigma.

### Quantification and Statistical Analysis

Western blots were quantified using ImageJ 1.48 software, downloaded from imagej.nih.gov/ij/. Data were analyzed using GraphPad Prism, version 6 (GraphPad Software, La Jolla, CA). All experiments are representative of at least 3 biological replicates, and data are presented as means ± standard deviation. Statistical significance was determined using one-way ANOVA. For multiple comparisons Dunnett’s test was performed. mRNA expression levels were assessed by two-tailed Student’s T-test using Microsoft Excel software. Significant P-values are as follows: *P ≤ 0.05, **P ≤ 0.01, ***P ≤ 0.001 (alpha: 0.05).

## Additional Information

**How to cite this article**: Fekrmandi, F. *et al.* The hormone-bound vitamin D receptor enhances the FBW7-dependent turnover of NF-κB subunits. *Sci. Rep.* **5**, 13002; doi: 10.1038/srep13002 (2015).

## Supplementary Material

Supplementary Information
