# Supplementary material for: The hormone-bound vitamin D receptor enhances the FBW7-dependent turnover of NF-κB subunits
Source: Sci Rep. 2015 Aug 13;5:13002. doi: 10.1038/srep13002 (PMC4534774; doi:10.1038/srep13002)
Supplement: Supplementary Information [file srep13002-s1.pdf]

The hormone-bound vitamin D receptor enhances the FBW7-dependent turnover of NF-κB subunits

Fatemeh Fekrmandi[1], Tian-Tian Wang[2], and John H. White[1, 2*]

Departments of Medicine[1] and Physiology[2], McGill University, Montreal, Quebec, Canada

*Address correspondence to john.white@mcgill.ca

Supplementary Figures.

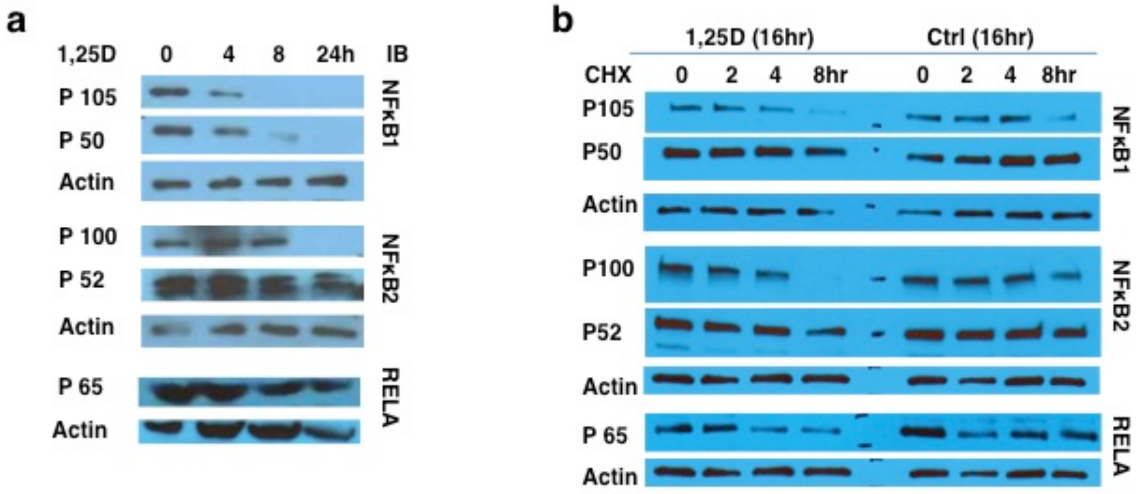

Supplementary Figure 1. a. Effect of treatment with 1,25D (100nM) on expression of NF-kB subunits in LNCaP cells. **b**. Effect of preincubation (16h) of LNCaP cells with DMSO vehicle (control) or 1,25D (100nM) followed by incubation with cycloheximide on turnover or NF-kB subunits, as indicated.

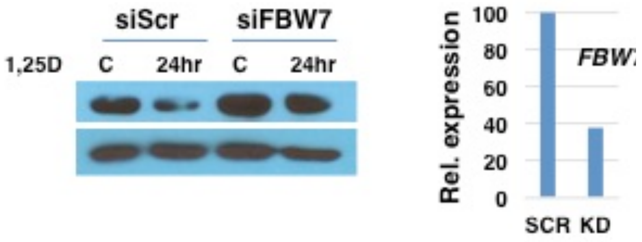

Supplementary Figure 2. Effect of treatment with 1,25D (100nM) on expression of NF-kb subunit p65 in LNCaP cells transfected with scrambled control siRNA or siRNA targeting *FBW7*. RT/qPCR analysis of *FBW7* expression is shown at right.

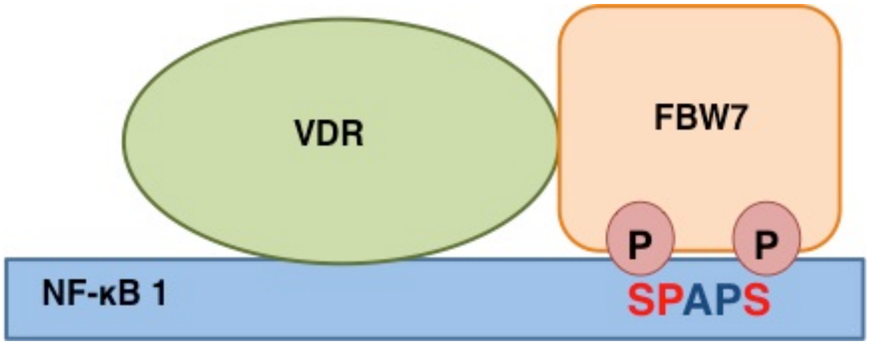

Supplementary Figure 3. Schematic illustration of suggested mechanism for formation of a VDR-FBW7-p105 complex to promote NF-κB degradation.

Supplementary Tables.

**Table 1: siRNAs**	
Gene name	siRNA Sequence
**FBW7**	5'-CCCTAAAGAGTTGGCACTCTA-3'
**Control**	5'-CAGGGTATCGACGATTACAAA-3'

**Table 2: qPCR primers**	
Primer name	Sequence
**FBW7- Forward**	5'-CAGCAGTCACAGGCAAATGT-3'
**FBW7- Reverse**	5'-GCATCTCGAGAACCGCTAAC-3'
**NFκB1- Forward**	GCAGCACTACTTCTTGACCACC
**NFκB1- Reverse**	TCTGCTCCTGAGCATTGACGTC
**NFκB2- Forward**	GGCAGACCAGTGTCATTGAGCA
**NFκB2- Reverse**	CAGCAGAAAGCTCACCACACTC
**RELA- Forward**	TGAACCGAAACTCTGGCAGCTG
**RELA- Reverse**	CATCAGCTTGCGAAAAGGAGCC
**NFκBIA- Forward**	TCCACTCCATCCTGAAGGCTAC
**NFκBIA- Reverse**	CAAGGACACCAAAAGCTCCACG
**MYB- Forward**	GGGAACAGATGGGCAGAAATCG
**MYB- Reverse**	GCTGGCTTTTGAAGACTCCTGC
**BCL3- Forward**	GAACACCGAGTGCCAAGAAACC
**BCL3- Reverse**	GCTAAGGCTGTTGTTTTCCACGG
**CCND1- Forward**	TCTACACCGACAACTCCATCCG
**CCND1- Reverse**	TCTGGCATTTTGGAGAGGAAGTG
**UCP2- Forward**	ACAAAGGGTTCATGCCCTCC
**UCP2- Reverse**	ACGAACATCACCACGTTCCA
**GCNT- Forward**	CTGGAAACGGAGAGGATGCCAT
**GCNT- Reverse**	CACGAAGTAGGCACTGCCAGAA
**GAPDH- Forward**	GTCTCCTCTGACTTCAACAGCG
**GAPDH- Reverse**	ACCACCCTGTTGCTGTAGCCAA
